# EBV Positive Diffuse Large B Cell Lymphoma and Chronic Lymphocytic Leukemia Patients Exhibit Increased Anti-dUTPase Antibodies

**DOI:** 10.3390/cancers10050129

**Published:** 2018-05-01

**Authors:** Marshall Williams, Maria Eugenia Ariza

**Affiliations:** 1 Department of Cancer Biology and Genetics, The Ohio State University College of Medicine, Columbus, OH 43210, USA; williams.70@osu.edu; 2 Institute for Behavioral Medicine Research, The Ohio State University College of Medicine, Columbus, OH 43210, USA

**Keywords:** Epstein-Barr virus (EBV), deoxyuridine triphosphate nucleotidohydrolase (dUTPase), Toll-like receptor 2 (TLR2), diffuse large B cell lymphoma (DLBCL), chronic lymphocytic leukemia (CLL)

## Abstract

The Epstein-Barr virus (EBV), which is a ubiquitous γ-herpesvirus, establishes a latent infection in more than 90% of the global adult population. EBV-associated malignancies have increased by 14.6% over the last 20 years, and account for approximately 1.5% of all cancers worldwide and 1.8% of all cancer deaths. However, the potential involvement/contribution of lytic proteins to the pathophysiology of EBV-associated cancers is not well understood. We have previously demonstrated that the EBV-deoxyuridine triphosphate nucleotidohydrolase (dUTPase) modulates innate and adaptive immune responses by engaging the Toll-Like Receptor 2 (TLR2), which leads to the modulation of downstream genes involved in oncogenesis, chronic inflammation, and in effector T-cell function. Furthermore, examination of serum samples from diffuse large B-cell lymphoma (DLBCL) and chronic lymphocytic leukemia patients revealed the presence of increased levels of anti-dUTPase antibodies in both cohorts compared to controls with the highest levels (3.67-fold increase) observed in DLBCL female cases and the lowest (2.12-fold increase) in DLBCL males. Using computer-generated algorithms, dUTPase amino acid sequence alignments, and functional studies of *BLLF3* mutants, we identified a putative amino acid motif involved with TLR2 interaction. These findings suggest that the EBV-dUTPase: TLR2 interaction is a potential molecular target that could be used for developing novel therapeutics (small molecules/vaccines).

## 1. Introduction

Epstein-Barr virus, which is a γ herpesvirus, is a ubiquitous virus that establishes a latent infection in over 90% of the global adult population. In addition to being the etiological agent of infectious mononucleosis (IM), it is implicated in several human malignancies including Burkitt’s lymphoma (BL), nasopharyngeal carcinoma (NPC), classical Hodgkin lymphoma (cHL), gastric cancer, and diffuse large B cell lymphoma (DLBCL) [1]. DLBCL is a heterogeneous disease that is classified based on micro-array-based gene expression profiling as germinal center B-cell like (GCB) DLBCL or activated B-cell-like (ABC) DLBCL [2]. A small percentage (10–20%) of DLBCLs are EBV-genome positive and such tumors are usually classified in the ABC DLBCL group [3]. While EBV-genome positive tumors were originally discovered in older (>50 years of age) immunocompetent individuals [4,5,6,7,8], they have recently been reclassified to EBV^+^ DLBCL-NOS due to the increased occurrence of EBV^+^ DLBCL in younger immunocompetent patients [9]. EBV is an independent factor that adversely affects risk and/or survival among patients with DLBCL [5,7]. Conversely, while EBV is not generally associated with developing chronic lymphocytic leukemia (CLL), which is the most common leukemia in adults in western countries [10], it is associated with Richter Syndrome (RS). RS, which occurs in 10% to 15% of patients with CLL, is a histological transformation to DLBCL resulting in a more aggressive lymphoma with a poorer prognosis [11,12,13,14]. While several studies have implicated EBV in RS [15,16], a mechanistic relationship has not been determined. However, a recent study demonstrated that therapy related to immunosuppression in patients with CLL resulted in EBV reactivation, which drove RS and the formation of the ABC subtype DLBCL in some patients [17]. This study as well as a recent study of DLBCL [18] has suggested that products of lytic EBV may contribute to the development of these malignancies, but additional studies need to be performed.

Studies to examine the roles of EBV-encoded proteins in cellular transformation have focused primarily on those proteins and RNAs expressed during latency. These studies have demonstrated unequivocally the roles of the latent membrane proteins LMP1 and LMP2A in the transformation process and the immunological response of the host to these proteins [19,20]. Until recently, there have been very few studies directed toward determining the role(s) of proteins expressed during the lytic replication of EBV in immune modulation or in transformation despite the fact that low levels of EBV reactivation and expression of genes associated with lytic replication are typically observed in a small number of cells in many tumors [21,22,23,24,25,26,27,28]. However, recent studies have demonstrated the expression of a large number of lytic genes in cell lines and, more importantly, in biopsy tissue [29,30,31]. It has been suggested that proteins encoded by these genes may contribute to EBV oncogenesis by modulating the tumor microenvironment through the release of growth factors and/or immunosuppressive cytokines [32] or more directly by inducing genomic instability [28]. Additional data obtained from studies using SCID and humanized mouse models support this premise [33,34,35,36].

The EBV gene *BLLF3* encodes for a deoxyuridine triphosphate nucleotidohydrolase (dUTPase), which is expressed during lytic/abortive lytic replication of the virus. While it has been difficult to quantify the amount of EBV-dUTPase present in tissue or serum because of the lack of sensitive assays, Ersing et al. [37] recently examined virus-host interactions during lytic replication using systemic proteomic quantitative analysis with tandem mass tags and mass spectrometry and estimated that the concentration of the EBV-dUTPase was 6000 nM and 7500 nM, respectively, in Akata and P3HR1 cells. There is indirect evidence to support the premise that EBV-encoded dUTPase is expressed and released from cells in vivo by following lytic and/or abortive replication. We have demonstrated, using quantitative real-time PCR, the expression of *BLLF3* in tumors (9/10) obtained from SCID mice injected with C666-1 cells, which is an EBV-genome positive NPC cell line [38]. Zhang et al. [39], using microarray technology, demonstrated the expression of *BLLF3* in PBMCs from a patient with acute phase IM and in EBV genome positive tumor cell lines established from patients with nasal NK/T-cell lymphoma. In addition, the EBV-encoded dUTPase protein has been detected using immuno-histochemical techniques in the upper epithelial layers of oral hairy leukoplakia (HL) lesions and the expression pattern was the same for BZLF-1 [40]. Similar results were obtained with lymphoid cells in tonsils from patients with IM and in NPC tissue [40,41]. Furthermore, we recently demonstrated by using immunohistochemistry the presence of the EBV- dUTPase in kidney biopsies from class III/IV Lupus nephritis (LN) patients. The EBV-dUTPase localized in infiltrating plasma-cell aggregates near glomeruli where neighboring cells expressing increased toll-like receptor 2 (TLR2) and IL-17 protein levels were observed, which suggests that EBV-dUTPase may exacerbate the immunopathologies in some LN patients [42]. We, as well as others, have demonstrated the presence of specific anti-EBV-encoded dUTPase antibodies in the sera of patients with IM, in reactivated and chronic EBV infections, in immunocompromised patients with HIV infections, and in immunocompetent patients with EBV genome positive diffuse large B-cell lymphoma, chronic lymphocytic leukemia and NPC [43,44,45], and unpublished data.

We have demonstrated that the dUTPases encoded by the human herpesviruses represent a new class of pathogen-associated molecular pattern (PAMP) proteins that have novel immuno-regulatory and neuro-regulatory functions, which may contribute to the pathophysiology of diseases caused by these viruses. Using the EBV-dUTPase as the prototype, our studies have demonstrated that it possesses novel functions independent of its enzymatic activity. Among them, the EBV-dUTPase acts as a trigger for TLR2, which leads to the activation of NF-κB and subsequent modulation of downstream genes involved in chronic inflammation and oncogenesis [46]. We have also demonstrated that these viral dUTPases are capable of differentially inducing the secretion of the pro-inflammatory T_H_1/T_H_17 cytokines IL-1β, IL-6, IL-8, IL-12p70, TNF-α, CCL20, and IFN-γ as well as the anti-inflammatory cytokine IL-10 in human primary immune cells [47,48,49,50,51]. Not only is CCL20 reported to promote cellular proliferation and differentiation of numerous cell types including malignant cells but IL-6, which is a positive regulator of CCL20, also functions as an autocrine growth factor for EBV-immortalized B-cells [52,53,54].

Since the interaction of EBV-dUTPase with TLR2 is the critical step for initiating the signaling cascade that leads to the establishment of a microenvironment that may support the survival and proliferation of EBV-transformed cells, the purpose of the present study was to elucidate the amino acid residues in the EBV-dUTPase important for interacting with TLR2.

## 2. Results

### 2.1. Identification of a Putative TLR2 Binding Motif within the EBV-dUTPase

**Figure 1:**
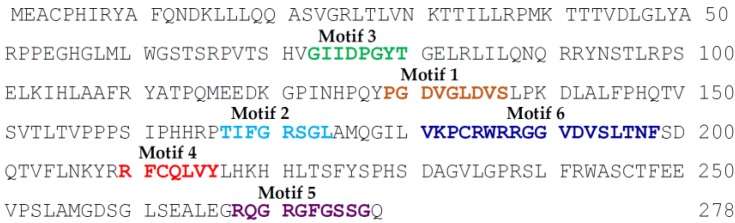
Epstein-Barr Virus deoxyuridine triphosphate nucleotidohydrolase (EBV-dUTPase) amino acid sequence. Typical dUTPase motifs 1–5 and the unique motif 6 characteristic of the Herpesviruses dUTPase family are depicted.

The EBV-encoded dUTPase is composed of 278 amino acids and, while it is the smallest of the human herpesviruses’ dUTPases, it contains all five motifs characteristic of dUTPases [55] as well as a unique motif (motif 6) found in herpesviruses’ dUTPases (see Figure 1) [56].

Using computer-generated algorithms (hydrophilicity, flexibility, mobility, solvent exposure, amphiphilicity, reverse turns, α-helical properties, and protrusion) to predict amino acid sequences that have the potential to interact with other proteins, we identified five sequences, which were then computer-ranked based upon their respective algorithms [51,57]. The amino acid sequences were 83–103 (rank 2), 109–140 (rank 1 contains the entire conserved motif 1), 174–194 (rank 4 contains most of motif 6, which is restricted to herpesviruses’ dUTPases), 210–237 (rank 5 contains conserved motif 4), and 253–276 (rank 3 contains conserved motif 5).

**Figure 2:**
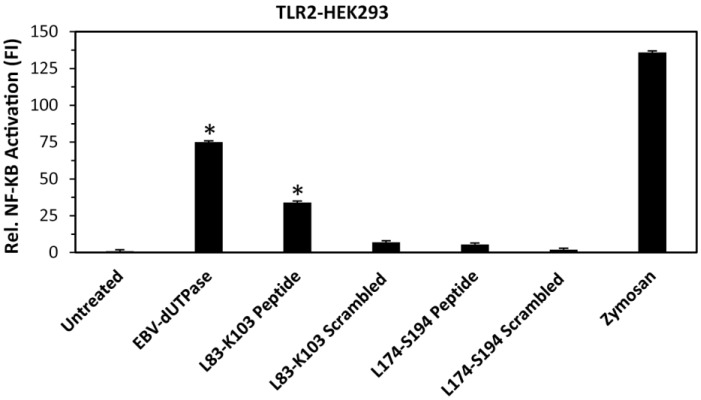
Activation of NF-κB by L83-K103 peptide in HEK293 cells stably expressing TLR2. Cells were transiently transfected with NF-κB luciferase reporter plasmid as we have described [40,43,44]. After 24–36 h, cells were treated with wild-type EBV-dUTPase, EBV-dUTPase peptide L83-K103, scrambled peptide L83-K103, EBV-dUTPase peptide L174-S194, scrambled peptide L174-S194 (10 μg/mL), zymosan (10 μg/mL), or left untreated for 8 h and luciferase reporter gene activity was measured. Values represent the mean fold induction (FI) ± SD relative to control (*n* = 3). Values represent the mean fold induction (FI) ± SD relative to control (*n* = 3). * *p* < 0.05 (Groups compared: wild-type dUTPase or synthetic peptide treated vs. untreated).

Since amino acid residues 83 to 103 were identified by computer-generated algorithms as the only non-conserved motif containing sequences with a high likelihood for interacting with other proteins and amino acid sequence 174 to 194 contains most of the unique motif 6 of unknown function, we next constructed synthetic peptides corresponding to amino acid residues leucine 83 through lysine 103 (L83-K103) as well as leucine 174 through serine 194 (L174-S194) and tested whether or not these peptides could induce NF-κB activation by engaging TLR2. As shown in Figure 2, only the L83-K103 peptide induced the transcriptional activation of the NF-κB reporter gene by 34-fold, which is approximately 50% of that exhibited by the full-length EBV-dUTPase protein (74-fold) while L174-S194 and corresponding scrambled control peptides did not cause a significant activation of NF-κB in human embryonic kidney 293 cells (HEK293)—stably expressing TLR2.

Further cytokine analysis of synthetic peptides in human PBMCs revealed that stimulation of cells with the EBV-dUTPase synthetic peptide L83-K103 but not scrambled peptide resulted in an increased production of IL-6 (9-fold increase over scrambled control peptide), IL-8 (2.5-fold increase), TNF-α (5-fold increase), IL-10 (4.8-fold increase), and IL-1β (3.3-fold increase) cytokines compared to untreated control cells. However, the cytokine response induced by L83-K103 peptide was not as strong as that observed in cells stimulated with the full-length EBV-dUTPase protein especially for IL-1β, TNFα, and IL-10 (see Table 1).

While the size and sequence homologies of the dUTPases encoded by members of the Herpesviridae Family vary considerably, a common feature in the dUTPases encoded by EBV, HSV-2, HHV-6, HHV-8, and VZV is their ability to trigger the activation of TLR2 [40,43,44]. Blast analyses of the amino acid sequences of the herpesviruses dUTPases as well as the human nuclear dUTPase demonstrated that the herpesviruses contained a sequence that was somewhat divergent especially with the β-herpesviruses that may represent a conserved sequence (see Table 2). 

**Figure 3:**
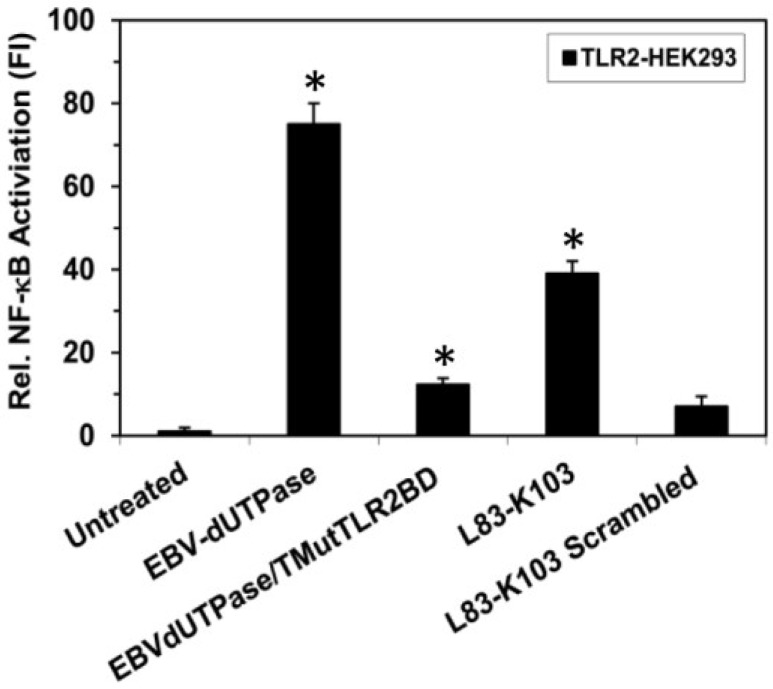
Site-directed mutagenesis of the putative TLR2 binding motif in the EBV-dUTPase inhibits NF-κB activation. TLR2-HEK293 cells were transiently transfected with NF-κB luciferase reporter plasmid as we have described [40,43,44]. After 24–36 h, cells were treated with wild-type EBV-dUTPase, a triple mutant (^82^ELR^84^ to ^82^GGG^84^) of the EBV-dUTPase TLR2 putative binding motif (EBVdUTPase/TMutTLR2BD), EBV-dUTPase peptide L83-K103, scrambled peptide L83-K103 (10 μg/mL) or left untreated for 8 h and luciferase reporter gene activity was measured. Values represent the mean fold induction (FI) ± SD relative to control (*n* = 3). * *p* < 0.05 (Groups compared: dUTPase treated vs. untreated and dUTPase triple mutant vs. wild-type dUTPase).

This TLR2 putative interactive motif is located in the β6 strand structure adjacent to motif 3, which is part of the catalytic site. This motif contains eight amino acid residues, which are included in the computer-generated algorithm sequence 83-103. The possibility of this motif being part of the TLR2 interactive domain is further supported by the studies described above, which demonstrate that a synthetic peptide corresponding to amino acids L83-K103 of the EBV-dUTPase induced NF-κB activation (see Figure 1) in TLR2-HEK293 cells and stimulated the secretion of pro-inflammatory cytokines in human PBMCs (see Table 2). To further confirm the importance of this motif for triggering TLR2 signaling, we generated a recombinant EBV-dUTPase protein containing a triple mutation (^82^ELR^84^ to ^82^GGG^84^; EBVdUTPase/TMutTLR2BD) and demonstrated that it significantly reduced NF-κB activation by 83% to 12.31-fold compared to the wild-type EBV-dUTPase protein (75-fold increase) (see Figure 3). Decreased cytokine secretion by stimulated PBMCs was also observed (data not shown), which highlights the importance of amino acid residues glutamate (E) 82, leucine (L) 83, and arginine (R) 84 in the activation of TLR2 signaling and further supporting this motif as a TLR2 putative interactive motif.

### 2.2. Anti-EBV-Encoded dUTPase Antibody in Patients with DLBCL and Chronic Lymphocytic Leukemia (CLL)

A better understanding of the diversity in the humoral response to EBV-dUTPase in health and disease states may enable us to identify EBV-dUTPase antibody patterns that could be used as markers for early diagnosis and/or to monitor treatment. Using our standard neutralization assay [44], we next conducted a pilot study to examine the humoral response to the EBV-dUTPase in healthy EBV carriers (*n* = 89) and in the B cell malignancies DLBCL (*n* = 36) and CLL (*n* = 66) sera samples from the European EPILYMPH case-control published study [58] exhibiting either a normal or abnormal/reactive antibody pattern to EBV, which was determined by de Sanjose et al. [58]. Sera from a control cohort of 431 individuals with no known health problems (268 females and 163 males) ranging in age from 18–92 years with a median age 63.38 ± 12.52 for females and 62.48 ± 13.46 for males, a DLBCL cohort of 36 cases (21 females and 15 males) ranging in age from 23 to 80 years old with a median age of 59.22 ± 18.39 and 49.40 ± 15.95 years, respectively, and a CLL cohort of 66 patients (33 females and 34 males) ranging in age from 30 to 87 years old with a median age of 69.52 ± 9.61 and 68.99 ± 11.75, respectively, were tested for the presence of anti-EBV dUTPase neutralizing antibodies. This study revealed an overall increase in neutralizing antibodies specific to the EBV-dUTPase in the case cohorts (36.11% and 39.39% for DLBCL and CLL, respectively) compared to the controls (12.76%) (see Table 3). Data analysis by gender shows a difference in the prevalence of dUTPase neutralizing antibodies between females and males within each cohort and across disease type relative to the controls. Within the DLBCL sera samples, there was a higher prevalence of dUTPase neutralizing antibodies in females than males (42.86% versus 26.66% in males). Additionally, the opposite was observed in the CLL cohort with males exhibiting a higher prevalence of dUTPase neutralizing antibodies than females (34.37% versus 44.12% in males). No differences were found within the control cohort between females and males, which suggests that the prevalence of dUTPase neutralizing antibodies in this group is independent of gender. More importantly, when comparing dUTPase antibody prevalence across disease type, it was found that the highest increase was observed in CLL male (44.12% versus 13.98% in controls) and DLBCL female cases (42.86% versus 11.67% in controls) (a 3.16-fold and 3.67-fold increase respectively), which were followed by the CLL female (34.37%, 2.95-fold increase) and DLBCL male cases (29.67%, 2.12-fold).

## 3. Discussion

EBV-associated malignancies are reported to account for approximately 1.5% of all cancers worldwide and represent 1.8% of all cancer deaths [59]. While most studies have focused on the contribution of EBV latency proteins such as LMP-1 and LMP-2A in oncogenesis, few studies have addressed the role, if any, that EBV proteins produced during lytic/abortive lytic replication may have in this process.

In the current study, we demonstrate the presence of increased neutralizing antibodies against the EBV-dUTPase in the sera of DLBCL and CCL patients from the EPYLYMPH study, which examined abnormal humoral responses to EBV [58]. In the EPYLYMPH study, the investigators reported that patients with aberrant EBV activity were identified by a broad immuno-reactive profile including antibodies to several peptides from proteins composing the Early Antigen diffuse (EA-D) complex (BMRF1, BALF2, BGLF5, and BXLF1) as well as EBNA1, VCA, and BZLF1 while uncomplicated carriers and sera from some patients with lymphomas exhibited a more restricted antibody pattern (EBNA1, VCA, and BZLF1). The dUTPase, which is encoded by the *BLLF3* gene, is an early protein that forms part of the EA-D complex. An important finding of our study is the observation that CLL male cases exhibited the highest prevalence of dUTPase neutralizing antibodies (44.12% versus 13.98% in controls) of all cases examined and had the lowest prevalence of an abnormal reactive antibody pattern to EBV. By contrast, CLL females had the highest increase in abnormal reactive antibody patterns to EBV (53.12% versus 22.22% in controls as determined by de Sanjose et al. [58]) but had the second lowest prevalence in dUTPase antibodies. Furthermore, within the DLBCL cohort, 67% (6/9) of female and all male sera samples (4) that tested positive for the presence of dUTPase neutralizing antibodies also expressed a normal/non-reactive antibody pattern to EBV. Overall, an increased prevalence of dUTPase neutralizing antibodies was consistently observed in sera of DLBCL and CLL patients who exhibited a normal/non-reactive antibody pattern to EBV. A recent study demonstrated that immediate early and early EBV proteins expressed during lytic replication of EBV are expressed in some tumor cells in patients with EBV^+^ DLBCL and that antibodies against these proteins are detected in patients’ sera. This led the investigators to propose that products from lytic/abortive lytic replication may contribute to tumor growth and survival [18]. Our previous studies have demonstrated that the EBV-dUTPase protein induces IL-6 in primary dendritic cells and PBMCs [46,47,48]. IL-6 is a growth factor for EBV-immortalized B cells and IL-6 over-expression has been shown to enhance growth of EBV-transformed lymphoblastoid cell lines (LCL’s) in SCID mice [60,61,62,63]. In addition, we have shown that EBV-dUTPase up-regulates the expression of CCL20 (335-fold) [49], which, in turn, may increase migration and trafficking of regulatory T cells (Tregs) into the tumor environment. Therefore, this dampens the immune response to EBV [54]. EBV-dUTPase also up-regulates the expression of BIC/miR155 [38], which is associated with aberrant inflammatory responses and oncogenesis, enhanced B-cell transformation, and the development of Tregs [64]. In all, these data support the premise that the dUTPase could modify the tumor microenvironment [32,38,65] and that the presence of antibodies directed against the dUTPase may be a useful marker for detecting aberrant virus replication in a subset of patients with DLBCL.

While several studies have demonstrated increases in EBV viral load, EBV miRNA and anti-EBV-antibodies in the sera of patients with CLL [66,67,68,69,70], the potential role of EBV in the development of CLL still remains poorly understood. Another study by de Sanjose et al. [58] reported that CLL samples exhibited the highest prevalence of abnormal anti-EBV antibody reactivity (40%) of any lymphomas examined. This finding was also observed in our analyses of anti-EBV dUTPase antibodies in the same samples. Two independent studies have recently demonstrated that a subgroup (53–59%) of patients presenting with CLL had significantly higher EBV-DNA copy numbers [69,70]. These patients required early treatment [69] and exhibited shorter survival rates [69,70]. It is also well documented that a small percentage (10–15%) of patients with CLL will undergo histological transformation into an aggressive form of DLBCL [11,12,13,14] referred to as Ritchter syndrome (RS). Recently, it was reported that RS might occur following aggressive therapy during CLL that results in the reactivation of EBV [17]. Therefore, it is possible that the CLL patients tested in this study and the study by de Sanjose et al. [58] represent two subgroups one in which EBV is a negative prognostic factor for patients with CLL and a second subgroup in which EBV contributes to RS transformation. While additional studies are necessary to delineate additional markers to distinguish these subgroups, the results of this study as well as that of de Sanjose et al. [58] suggest that antibodies against EBV-dUTPase and EA-D, which the dUTPase is a component of, could be useful markers for initially identifying such patients.

There is a growing body of evidence demonstrating that the reactivation of latent herpesviruses, as indicated by higher antibody titers to proteins expressed during lytic or abortive-lytic replication, occurs when the immune system is compromised [45,48,71,72,73,74]. Several studies on EBV have established that reactivation of the virus usually results in abortive-lytic replication in which only immediate early and early genes are expressed and, therefore, no new virus is produced [75,76,77]. Since the dUTPase is expressed as an early protein, this would suggest that abortive and/or lytic replication occurs in a subset of patients with DLBCL and CLL.

We have previously demonstrated that the EBV-dUTPase triggers NF-κB activation by engaging TLR2 homodimers [46] while the dUTPases encoded by HSV-2, HHV-6, HHV-8, and VZV require ligation of the TLR2/1 heterodimer complex to activate NF-κB [50]. Follow-up studies demonstrated that these viral dUTPases are capable of differentially inducing the secretion of the pro-inflammatory T_H_1/T_H_17 cytokines IL-1β, IL-6, IL-8, IL-12p70, TNF-α, and IFN-γ as well as the anti-inflammatory cytokine IL-10 in human primary immune cells [46,47,48,49,50,51]. This suggests that they can modulate the cellular microenvironment [32,38,65]. While sequence analyses have demonstrated that the α and γ herpesvirus members contain the five highly conserved motifs characteristic of the homotrimeric and monomeric dUTPases, members of the β herpesvirus group do not. However, all the human herpesviruses contain an additional conserved motif (domain 6) that is absent in the homotrimeric dUTPases [56]. It has been suggested that this novel herpesvirus-specific domain may contribute to some unknown novel function. With the exception of the EBV-dUTPase [55], no crystal structure data is available for the other human herpesviruses’ dUTPases. The results shown in this study demonstrate that amino acid residues between ^81^G to ^103^K of the EBV-dUTPase are important for binding to and activating TLR2 signaling. This TLR2 putative interactive motif is located in the β6 strand structure adjacent to motif 3, which is part of the catalytic site.

The lack of control of EBV abortive/lytic replication may reflect a variety of physiological processes including stress and aging, which affect T-cell function and, therefore, the role of EBV in oncogenesis may or may not be a direct effect. However, our results suggest that lytic/abortive lytic replication occurs in patients with DLBCL as well as CLL. The EBV-dUTPase may alter the tumor microenvironment [32,38,65] by providing a selective advantage (growth/survival) to the malignant cell. Future studies using a larger cohort of patients will be necessary to determine whether there is a possible relationship between EBV-dUTPase expression and malignant progression as well as whether or not the presence of anti-EBV-dUTPase antibodies could be useful for diagnostic purposes. While additional experiments involving crystal structures of TLR2: EBV-dUTPase complexes are needed to confirm the specific amino acid residues of the EBV-dUTPase that interacts with TLR2, the results from the current study support the premise that the EBV-dUTPase-TLR2 interaction could be used as a target for developing novel therapeutics specifically small molecules and/or vaccines.

## 4. Materials and Methods

### 4.1. Construction of EBV-dUTPase Triple Mutant

EBV-dUTPase containing a triple point mutation E82G, L83G, and R84G (^82^ELR^84^ to ^82^GGG^84^) was generated by site-directed mutagenesis using the QuikChange Lightning Mutagenesis system (Stratagene, Santa Clara, CA, USA), which was previously described [78] and the primer set: Forward: 5′-CCGGTCACGTCTCATGTTGGCATCATCGATCCCGGCTACACG-3′; Reverse: 5′-CGTGTAGCCGGGATCGATGATGCCAACATGAGACGTGACCGG-3′. The PCR conditions used include one cycle at 95 °C for 2 min, which was followed by 18 cycles of 95 °C for 20 s, 60 °C for 10 s, and 68 °C for 2.5 min, and one cycle at 68 °C for 5 min. Amplified products were DpnI digested and screened for the β-galactosidase (β-gal+) phenotype. DNA was then purified and the amino acid changes E82G, L83G, and R84G were verified by using sequence analysis.

### 4.2. Peptide Synthesis

EBV-dUTPase peptides ^83^LRLILQNQRRYNSTLRPSELK^103^, ^174^LAMQGILVKPCRWRRGGVDVS^194^ as well as the respective scrambled controls peptides ^83^ELQPKRTLQSRLYRINLSNRL^103^ and ^174^KRLGVCIQWVGLPRDVMRSAG^194^ were synthesized in house in the Peptide Protein Engineering Laboratory at the Tzagrournis Medical Research Facility at The Ohio State University. Peptide synthesis was performed on a Milligen/Biosearch 9600 solid-phase peptide synthesizer (Bedford, MA, USA) using Fmoc/t-But chemistry. The C-terminal amino acid loaded on CLEAR ACID resin (0.32 mmol/gm) (e.g., in case of peptide WILL 83-103, Fmoc-Ile-CLEAR ACID Resin (Peptides International, Louisville, KY, USA) was used for the synthesis. All peptides were cleaved from the resin using cleavage reagent B (Trifluoroacteic acid:Phenol:Water:Triisopropyl silane 90:4:4:2) and crude peptides were purified on preparative Reverse Phase-High Pressure Liquid Chromatography (RP-HPLC) using Vydac C-4 column and the acetonitrile-water (0.1% TFA) gradient system. All fractions were analyzed on analytical RP-HPLC and characterized by using Matrix Assisted Laser Desorption Ionization mass spectroscopy (MALDI) at The Ohio State University Campus Chemical Instrumentation Center. RP-HPLC fractions showing the same mass spectrum peak were pooled together and lyophilized. The respective scrambled peptides were synthesized using the number of amino acids present in natural sequences and were scrambled manually. All pure peptides were further characterized using MALDI mass spectroscopy analysis to confirm the calculated and observed molecular weight, which include L83-K103 (M+H+) Cal/Obs 2598.49/2598.47, SCRL83-K103 (M+H+) Cal/Obs 2598.49/2598.23; L174-S194 (M+H+) Cal/Obs 2341.82/2341.13, SCRL174-S194 (M+H+) Cal/Obs 2341.84/2341.42.

### 4.3. Purification of Recombinant EBV-dUTPase Protein

Sub-cloning and purification of recombinant EBV-dUTPase mutant and wild-type proteins were performed as previously described [40,41,44]. All recombinant dUTPase protein preparations were tested for the presence of contaminants, which was described previously [40,41,44], and were free of detectable levels of LPS, peptidoglycan (SLP-HS), DNA, or RNA. Protein concentration was determined using the Qubit fluorimeter (Invitrogen, Carlsbad, CA, USA). The purified recombinant dUTPase proteins used in these studies were stored at −80 °C until further use.

### 4.4. EBV-dUTPase Neutralization Assays

Neutralization assays for the EBV dUTPase were performed as previously described [44]. In brief, 5 µL of human serum were mixed with 5 µL of purified EBV-dUTPase (3–5 units of enzyme) for 30 min at room temperature prior to assaying for enzymatic activity. EBV-dUTPase activity was determined as described previously [44]. For positive controls, assays were performed in the presence of human serum that lacked detectable antibodies to the EBV. Negative controls were also performed in the absence of the enzyme preparation. A unit of EBV-dUTPase activity was defined as the amount of enzyme required to convert 1 nmole of dUTPase to dUMP and pyrophosphate/min/mL of enzyme at 37 °C. Units of enzymatic activity neutralized per mL of serum were obtained as follows: (Ucontrol–Userum). Serum with neutralizing units greater than or equal to two standard deviations from the control were considered “positive” for dUTPase neutralizing antibodies.

### 4.5. Patients

The patient samples in this study were collected from 1988–2003 as part of the EPILYMPH case-control study carried out in six European countries by de Sanjose et al. [58]. Cases were defined as all consecutive patients having their initial diagnosis of lymphoid malignancy during the study period. The diagnosis of lymphoma was verified by histology and 99% of cases were supplemented by immunohistochemistry tests and flow cytometry. The cases were categorized according to the World Health Organization (WHO) Classification for Neoplastic Diseases of the Lymphoid Tissues and included all B-cell, T-cell, and NK-cell neoplasms as well as Hodgkin’s lymphoma [79]. Additionally, 20% of all diagnosed cases in each country were externally reviewed by a panel of international pathologists. The panel diagnosis is the one used in this analysis in the rare circumstance of disagreement between the local and the panel pathologists. Subjects with a diagnosis of uncertain malignant potential such as post-transplant lymphoproliferative disorder or monoclonal gammopathies of undetermined significance were excluded. Immunosuppressed patients were excluded from the analysis.

### 4.6. Cell Culture

Human embryonic kidney (HEK293) cells stably expressing human TLR2 (TLR2-HEK293; Invivogen, San Diego, CA, USA) were maintained in DMEM-supplemented medium, as recommended by the manufacturer [46,49,50,78]. Human peripheral blood mononuclear cells (PBMCs) from healthy subjects were obtained from Astarte Biologics (Bothell, WA, USA). 

### 4.7. Luciferase Reporter Gene Assays

HEK293 cells (2.5 × 10^5^) were seeded into 12-well plates and 24 h later transiently transfected with pNFκB-Luc, pRL-TK reporter vectors (Promega, Madison, WI, USA), or with empty vectors as described [46,49,50,78]. About 24 h to 36 h after transfection, cells were treated with recombinant wild-type or truncated EBV dUTPase proteins (10 μg/mL), zymosan (10 μg/mL; positive control for TLR2 activation) for 8 h or left untreated. After treatment, cell lysates were prepared and reporter gene activities were measured using the dual-luciferase reporter assay system (Promega). Data were normalized for transfection efficiency by measuring Renilla luciferase activity and expressed as mean relative stimulation ± SD.

### 4.8. Cytokine Profile Induced by Herpesviruses-Encoded dUTPases

PBMCs were seeded at a density of 2.5 × 10^5^ in 24-well plates and cultured in AIM-V serum-free medium supplemented with l-glutamine (2 mM), streptomycin (50 μg/mL), and gentamycin (10 μg/mL). The next day, cells were stimulated with wild-type or truncated dUTPases (10 μg/mL), EBV-dUTPase peptide L83-K103, scrambled peptide L83-K103 or left untreated for 24 h. Following treatment, cell culture supernatants were collected and cytokine levels were measured by using ELISA (MSD Multi-array and Multi-spot human cytokine kit), which we described previously [46,49,50,78]. Concentrations are expressed as pg/mL and represent the mean ± SD of an *n* of 3.

### 4.9. Statistical Analysis

Statistical analyses were performed using a paired two-sample t-test for the means and *p* values were reported when displaying a significant value (*p* < 0.05). Values represent the mean ± SD of at least three independent experiments.

## 5. Conclusions

Examination of serum samples from diffuse large B-cell lymphoma (DLBCL) and chronic lymphocytic leukemia (CLL) patients revealed the presence of increased anti-dUTPase neutralizing antibodies in both cohorts compared to controls with the highest levels (3.67-fold increase) observed in DLBCL female cases and the lowest (2.12-fold increase) in the DLBCL males. Furthermore, using computer-generated algorithms, dUTPase amino acid sequence alignments, and functional studies of *BLLF3* mutants, we identified a putative amino acid motif involved with TLR2 interaction and demonstrated that amino acid residues between ^81^G to ^103^K of the EBV-dUTPase are important for binding to and activating TLR2 signaling. These findings suggest that the EBV-dUTPase: TLR2 interaction is a potential molecular target that could be used for developing novel therapeutics (small molecules/vaccines).

## Figures and Tables

**Table 1:** Cytokine profile induced by EBV-dUTPase peptide L83-K103 in human ^a^ PBMCs at 48 h.

Treatments (10 µg/mL)	IL-6 (pg/mL)	IL-1β (pg/mL)	TNFα (pg/mL)	IL-8 (pg/mL)	IL-10 (pg/mL)
Untreated	5 ± 0.8	31 ± 22.8	5 ± 4.9	407 ± 5.7	8 ± 3.8
EBV-dUTPase	9570 ± 5.7	978 ± 15	379 ± 123	35,039 ± 219	311 ± 35
Scrambled peptide L83-K103	369 ± 312	34 ± 24.4	9 ± 6.2	8738 ± 267.6	9 ± 5.2
EBV-dUTPase peptide L83-K103	3272 ± 6	111 ± 21.5	45 ± 6.6	21,934 ± 14	43 ± 6.2

^a^ PBMCS from healthy donors were treated with EBV-dUTPase full-length protein, EBV-dUTPase peptide L83-K103, scrambled control peptide (10 µg/mL), or left untreated for 48 h. Culture supernatants were collected for cytokine analysis by ELISA. Cytokine levels are expressed as pg/mL. Values represent mean ± SD of an *n* = 3.

**Table 2:** TLR2 putative binding motif.

dUTPases	Amino Acid Sequence
EBV	^81^GELRLILQNQ^90^
HHV-8	^109^GEIQVILLNK^118^
HSV-1/2	^102^GTVMAVVAP^110^
VZV	^130^GVISALLYYR^139^
HHV-6A	^207^TDISVFLMNL^116^
HHV-7	^215^NVISISLINL^224^
HCMV	^173^LQVPQLDVVNL^183^
Human	^84^GNVGVVLFNF^93^

**Table 3:** Detection of anti-EBV-dUTPase antibodies (Ab) in patients with DLBCL or CLL.

Clinical Status	Gender	% Positive EBV dUTPase Ab ^a^	% Abnormal Reactive Ab Pattern to EBV (ARP_EBV) ^b^	% ARP_EBV & dUTPase Seropositive ^c^
Controls	Females	11.67 (32/268)	22.22 (10/45)	8.88 (4/45)
	Males	13.98 (23/163)	11.36 (5/44)	4.54 (2/44)
DLBCL	Females	42.86 (9/21)	33.33 (7/21)	14.28 (3/21)
	Males	26.66 (4/15)	20.00 (3/15)	0.00 (0/15)
CLL	Females	34.37 (11/32)	53.12 (17/32)	18.75 (6/32)
	Males	44.12 (15/34)	20.59 (7/34)	14.70 (5/34)

^a^ dUTPase neutralizing assays were performed as described previously [44]. Values in parentheses represent the number of positive sera in either cases or controls/total sera. The total number of control sera (*n* = 431) include 89 samples from the EPILYMPH case-control study [58] as well as 352 samples from other published studies [44,45,48]. ^b^ Individuals’ sera exhibiting an abnormal reactive Ab pattern to EBV (ARP_EBV) was determined by de Sanjose et al., as part of the EPILYMPH case-control previously published study [58]. Values represent the percentage of sera samples exhibiting EBV-IgG reactivity to combined immuno-dominant epitopes of EBNA1 and VCA-p18-based ELISA assays and abnormal reactivity/intensity score on immunoblots to EBV antigens (ex: EAd-p47/54, EAd-p138) other than/besides EBNA1, VCA-p40, VAC-p18, and ZEBRA predominantly recognized by healthy EBV immunocompetent individuals [58]. Values in parentheses represent the number of individuals exhibiting increased/abnormal Ab responses to EBV proteins in either cases or controls sera/total sera. ^c^ Values represent the percentage of sera samples that were positive for anti-EBV-dUTPase antibodies and also had increased/abnormal EBV reactivity. Values in parentheses represent the number of positive sera in either case or controls/total sera.

## Abbreviations

The following abbreviations are used in this manuscript:

TLR2	Toll-like receptor 2
DLBCL	Diffuse large B cell lymphoma
CLL	Chronic lymphocytic leukemia
EBV	Epstein-Barr virus
dUTPase	Deoxyuridine triphosphate nucleotidohydrolase
HSV-2	Herpes simplex virus type 2
HHV-6	Human herpesvirus 6
HHV-8	Human herpesvirus 8
VZV	Varicella-zoster virus
HEK293	Human embryonic kidney cells
PBMCs	Peripheral blood mononuclear cells
